# Physical Fitness Tests as Predictors of High-Intensity Running Performance in Rugby

**DOI:** 10.3390/sports11080156

**Published:** 2023-08-16

**Authors:** Takashi Sato, Minas Nalbandian, Masaki Takeda

**Affiliations:** 1 Graduated School of Health and Sports Science, Doshisha University, Kyoto 610-0394, Japan; cyhc0002@mail4.doshisha.ac.jp; 2 School of Medicine, Stanford University, Stanford, CA 94306, USA; mnalband@stanford.edu; 3 Faculty of Health and Sports Science, Doshisha University, Kyoto 610-0394, Japan

**Keywords:** repeated sprint ability, rugby union, high-intensity running

## Abstract

Understanding the physical fitness elements that influence high-intensity running ability during rugby matches is crucial for optimizing player performance and developing effective training strategies. In this study, we aimed to investigate the relationships between various physical fitness components and high-intensity running ability in rugby. For this purpose, 60 Japanese university rugby players were randomized into four groups and two matches were played. The participants were monitored in two matches, and their running abilities were assessed using GPS sensors. The running time was divided into three running velocity categories: distance run at ≤5.4 km/h (low-intensity running); distance run at 5.5~17.9 km/h (medium-intensity running), and distance run at ≥18.0 km/h ≤(high-intensity running) and backs and forwards were evaluated separately. To determine which physical fitness test is more predictive of performance, we decided to correlate several physical test performances with the running time intensities during the matches. Independently of the position, the high-intensity running time correlated with the repeated sprint ability (RSA) and the 40 m sprint speed. The results suggest that RSA measured in the field is the most important high-intensity running ability predictor during a match for both positions.

## 1. Introduction

Running performance, including distance, speed, and acceleration, significantly impacts the overall team performance in field sports. The Rugby Union has undergone rule changes in recent years to enhance the match’s appeal and increase its pace [1,2]. A typical rugby match involves collisions and intermittent exercise, with players covering 5–7 km in an 80 min game [3,4]. Crucial moments in the match often involve high-intensity running, such as sprints, which can determine the outcome [5].

Research indicates that forwards (FWs) cover an average high-intensity distance of 462 m, while backs (BKs) cover 655 m during actual matches [6]. Forwards typically perform 15–17 high-intensity runs lasting 45–52 s, while backs make 7–16 runs lasting 26–28 s [7]. Notably, high-intensity running declines in the second half of matches [8]. Enhancing high-intensity running ability throughout the match can elevate the competitiveness of rugby matches. Thus, it is important to identify key physical fitness factors that influence players’ high-intensity running ability during rugby matches.

Running performance tests, particularly sprint and endurance tests, are commonly used to assess physical fitness in rugby [9,10] due to their significant impact on match performance. However, these tests do not reflect the dynamic of the sport. The repeated sprint ability (RSA) test, which combines sprinting and endurance, is utilized in training for sports involving intermittent exercise [11,12,13]. The RSA test measures the ability to repeatedly perform high-intensity running at speeds of at least 90% maximum power [2]. While field-based RSA tests are considered good indicators of running ability during matches [12], their validation for high-intensity running (Dist. Z46) in actual matches is still needed.

In this study, we aimed to establish which test can better correlate with performance in the field. To achieve this objective, 60 Japanese university rugby players were randomly divided into four groups based on their positions as backs and forwards, as well as their repeated sprint ability. The participants were closely monitored during two matches, with their running abilities assessed using GPS sensors. The running time was categorized into three distinct levels: distance covered at speeds of ≤5.4 km/h (low-intensity running), distance covered at speeds ranging from 5.5 to 17.9 km/h (medium-intensity running), and distance covered at speeds ≥ 18.0 km/h (high-intensity running). To ascertain the most reliable physical fitness test for predicting performance, our study sought to establish correlations between various physical test performances and running time intensities during rugby matches. Our results suggested that the RSA test was the more predictive test for the performance in the field for back and forward players.

## 2. Materials and Methods

### 2.1. Subjects

In this study, a total of sixty Japanese university-level rugby players with an average age of 20.5 ± 0.7 years were recruited. The subjects had played rugby for 7–10 years.

Among the initial sixty subjects, eight were excluded from the analysis due to injuries sustained during the matches, resulting in a final sample size of fifty-two individuals (twenty-three backs and twenty-nine forwards). To ensure the completion of the matches, substitute players were allowed to participate, although they were not included in the subsequent statistical analysis. This study was approved by the Doshisha University Code of Research Ethics based on an ethics review committee (application number 18036). The subjects expressed their consent to participate in this study.

### 2.2. Study Design

The participants underwent various physical assessments and fitness tests, including evaluations of body composition, sprint ability, endurance capacity, repeated sprint ability (RSA), muscle strength, and power. Detailed descriptions of each test are provided below.

After completing the assessments, the subjects were randomly divided into four groups (A, B, C, and D). When randomizing the subjects in this study, to ensure that the rugby levels of all four groups were equal, subjects were divided not only by RSA and position but also by their level of rugby skill (evaluated by the coach). Therefore, the authors assume almost no difference in rugby level among the four groups. In addition, since the subjects in this study were from the same team, they regularly trained based on similar daily tactics. In each game of the experiment, the subjects were instructed to replicate their daily training tactics without any extreme tactical differences in the tactics of each game. Thus, the experimental design was designed to eliminate as much as possible the differences between the groups, not only at the rugby level but also in tactics, so that the relationship between high-intensity running ability and physical fitness characteristics could be extracted.

In all 4 groups, subsequently, two matches were played: A vs. B and C vs. D, both lasting eighty minutes. Throughout these matches, the players’ running performance was measured using GPS devices (further details provided later).

### 2.3. Measurements in Physical Characteristics and Physical Fitness

#### 2.3.1. Body Composition

Height was measured using a YS silver studio meter. A multi-frequency body composition monitor (Tanita MC-780-N.Tanita, Tokyo, Japan) was used to measure weight, body fat (%Fat), and muscle mass (%Muscle). %Fat was calculated by dividing the fat mass by body mass. Likewise, %Muscle was calculated by dividing muscle mass by body mass. The body mass index (BMI, BK: 26.9 ± 1.3; FW: 31.1 ± 2.5) was calculated by dividing body mass by the height squared.

#### 2.3.2. Forty-Meter Sprint (40 m Sprint)

A phototube sensor (TAG Heuer) was used. Measurements were taken twice, and the better time was used for the analysis.

#### 2.3.3. Twelve-Minute Run (12 min Run)

The course was a 350 m tartan track, in which markers were placed at 5 m intervals. If a runner stopped between markers at the end of the 12 min, the distance run was rounded down to the last marker.

#### 2.3.4. Repeated Sprinting Ability (RSA) Test

**Figure 1:**
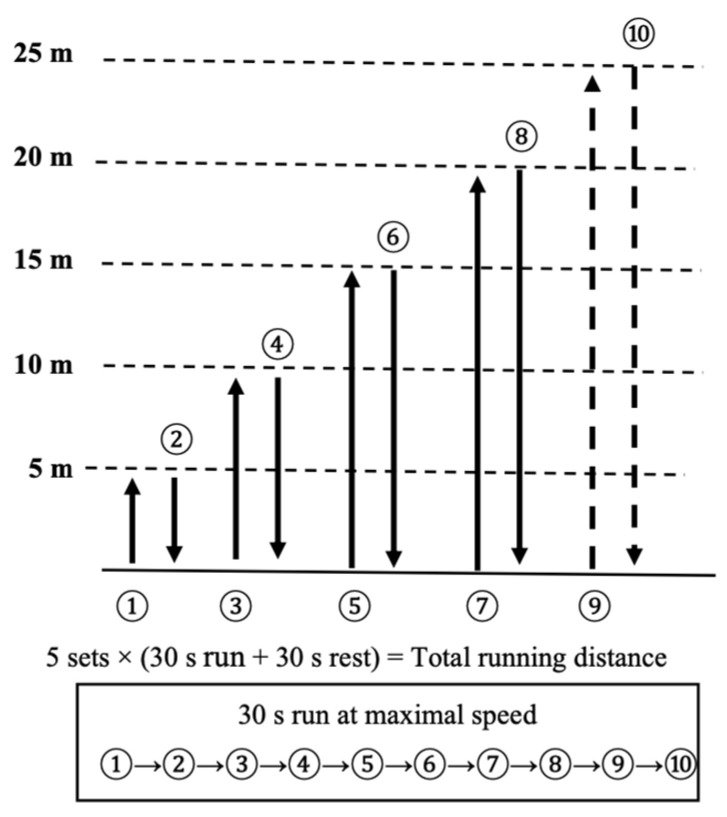
The repeated sprint ability (RSA) test. RSA was defined as the total distance ran after 5 sets of 30 s at maximum speed with 30 s of resting time.

In our study, players underwent a high-intensity exercise protocol, running back and forth at maximum speed for 30 s at various distances, including 5 m, 10 m, 15 m, 20 m, and 25 m. The exercise routine consisted of 5 sets, with each set separated by 30 s of rest (as shown in Figure 1).

Throughout the test, we recorded the total running distance covered by each player for the entire duration of 30 s per set, summing up to a cumulative running time of 150 s over the 5 sets. This approach allowed us to assess the players’ performance under different sprinting distances and observe any potential variations in their running capabilities across these intervals.

By employing this structured exercise regimen and recording the total running distance achieved by the players, we sought to gain insights into their speed, endurance, and anaerobic capacity.

#### 2.3.5. Lactate Measurement

To measure the blood lactate, 2 μL of blood was collected from the fingertips after 5 min of rest before and immediately after the RSA test and analyzed for blood lactate using a Lactate Pro II (Arkray, Kyoto, Japan).

#### 2.3.6. Countermovement Jump (CMJ)

A multi-jump tester (DKH Co., Ltd., Tokyo, Japan) was used to measure countermovement jump. The subjects were asked to place their hands over the waist to control trunk movement. Then, a countermovement jump was performed twice, and the height was measured each time. The highest value of the two tests was used for the analysis.

#### 2.3.7. Bench Press

To measure upper body strength, a bench press maximum repetition was tested. The test consisted in having the participants facing up on a bench, with both feet placed flat on the floor in the supine position, and pushing a barbell with weight with both arms, with a hands’ separation width on the bar such that the elbows were bent at right angles and the upper arms were parallel to the bar. The maximum lift was measured.

#### 2.3.8. Squat

To measure the leg strength, a squat maximum repetition was evaluated. The test consisted in having the participants with the barbell resting on the rear of the deltoid muscles of both shoulders behind the neck and the trapezius muscle lower the hips from the upright posture (both knees fully extended) to the same height as the knees and then return to the upright position. The maximum lift was measured.

#### 2.3.9. Clean

To measure the participant’s power, a Clean maximum repetition was evaluated. The test consisted in having the participants start in a squatting position, gripping the barbell with hands wider than shoulder-width apart. With a swift pull, the participants were asked to extend their hips, knees, and ankles, propelling the barbell upward while keeping it close to the body. As the barbell reached its maximum height, the participants quickly dropped into a front squat position and caught the barbell at shoulder level, simultaneously transitioning from the pull to the catch phase. The maximum lift held steady for 3 s or more was recorded.

#### 2.3.10. Running Performance in a Match

To measure the running distances and velocities during a match, a GPS sensor (GPSPORT evo, GPSPORT; size: 63.6 mm × 43.7 mm × 18.5 mm, weight approx. 60 g, measuring frequency 10 Hz) was attached to the upper back of worn vests to monitor running performance during a match. The items measured by the GPS sensor were as follows.

Dist. (m): total distance run at all speeds.
Dist. Z1 (m): distance run at ≤5.4 km/h (low-intensity running).
Dist. Z23 (m): distance run at 5.5~17.9 km/h (medium-intensity running).
Dist. Z46 (m): distance run at ≥18.0 km/h ≤(high-intensity running).
Max speed (m/s): maximum speed during the match.
Acc. (frequency): number of events with accelerations ≥ 2.5 m/s^2^.

It has been reported that the threshold of the velocity of high-speed running during rugby matches is 5 m/s, i.e., 18 km/h. Therefore, we defined high-intensity running (Dist. Z46) as over 18.0 km/h [14].

### 2.4. Statistics

An unpaired *t*-test was used to compare the physical characteristics, fitness, and running performance during a rugby match between BKs and FWs. Pearson’s correlation analysis was used for the correlations among physical characteristics, fitness, and running ability during matches in BKs and FWs, respectively. For those items which showed a significant correlation among physical characteristics, fitness, and running performance during a match, a partial correlation analysis with physical characteristics (height and weight, %Fat, %Muscle, and BMI) as an adjustment variable was performed to further remove the influence of items that were considered to influence each other. Excel Statistics 2019 was used for the statistical analysis and statistical significance was set at *p* ≤ 0.05.

## 3. Results

To study and compare the physical fitness of back (BK) and forward (FW) rugby players, sixty university rugby players were recruited. Eight players were excluded from the analysis due to injuries sustained during the matches, resulting in a final sample size of fifty-two individuals (twenty-three backs and twenty-nine forwards). The participants’ physical characteristics, fitness, and running ability in two matches were studied (Table 1).

FW players showed significantly higher height, weight, %Fat, %Muscle, and BMI. BK players showed significantly higher running ability such as in 40 m sprint, 12 min run, RSA, and CMJ. As supposed, muscle strength measured as the maximal value of squat and bench press was significantly higher in FWs compared to BKs.

To quantify the physical performance during the two matches, the participants were monitored with a GPS. Running velocities were recorded and classified into different categories, where Dist. (m), the total distance run at all speeds; Dist. Z1 (m), the distance run at ≤5.4 km/h (low-intensity running); Dist. Z23 (m), the distance run at 5.5~17.9 km/h (medium-intensity running); Dist. Z46 (m), distance run at ≥18.0 km/h ≤ (high-intensity running); Max. speed (m/s), maximum speed during the match; Acc. (frequency), number of events with accelerations ≥ 2.5 m/s^2^. Comparative analysis showed that all of the measurements for the running ability during matches (Dist., Dist. Zi, Dist. Z46, Max. speed, and Acc.) were significantly higher in BKs than in the FW participants (Table 1).

High-intensity running ability (Dist. Z46) is considered the most important running parameter to determine performance during a rugby game. To identify which physical characteristics and fitness factors reflect high-intensity running ability (Dist. Z46) during matches, we conducted a correlation analysis among physical characteristics, physical fitness, and running ability in matches. Correlation coefficients among measured items are shown in Table 2 for BKs and in Table 3 for FWs.

**Figure 2:**
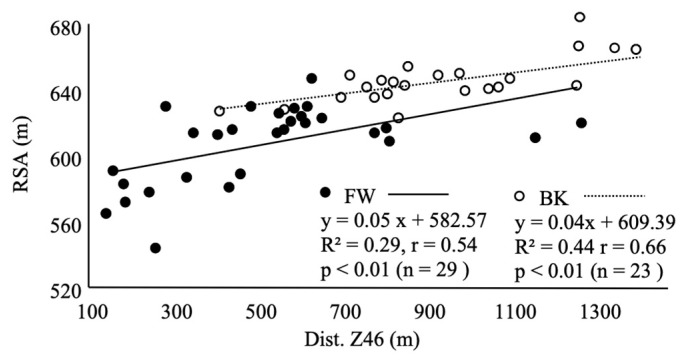
Correlation between Dist. Z46 and repeated sprint ability (RSA).

**Figure 3:**
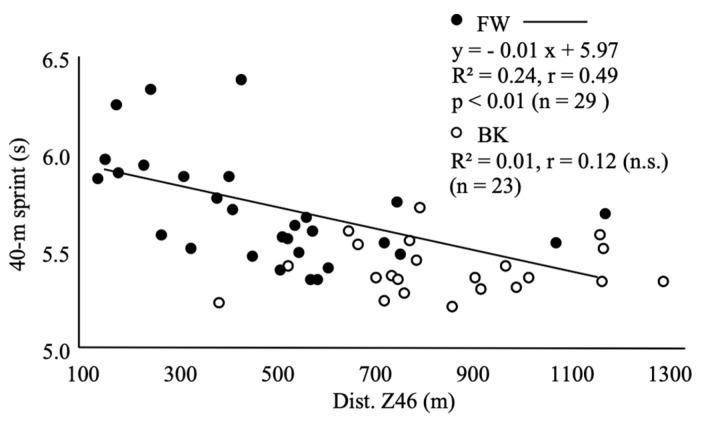
Correlation between Dist. Z46 and 40 m sprint.

**Figure 4:**
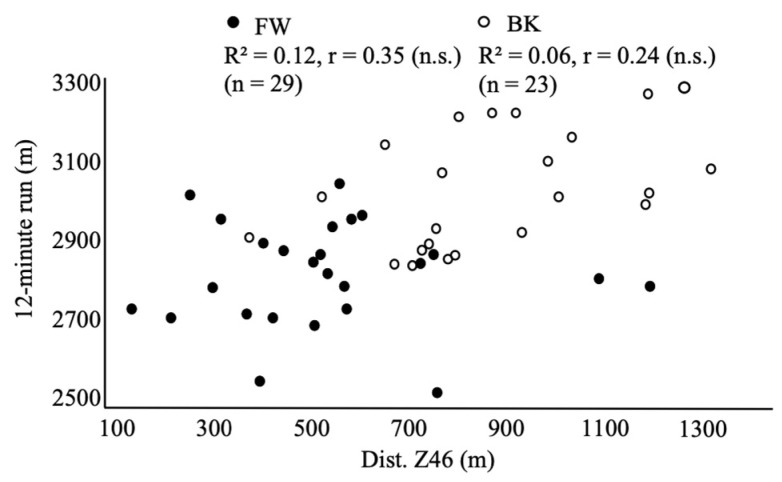
Correlation between Dist. Z46 and 12 min run.

For BKs, %Fat (r = −0.47, CI = −0.74 to 0.07), %Muscle (r = 0.46, CI = −0.73 to 0.06), and RSA (r = 0.66, CI = 0.34 to 0.84) significantly correlated with Dist. Z46 in matches. For FWs, body mass (r = −0.67, CI = −0.83 to −0.40), %Fat (r = −0.42, CI = −0.68 to −0.06), %Muscle (r = 0.59, CI = 0.29 to 0.79), BMI (r = −0.62, CI = −0.80 to −0.32), RSA (r = 0.54, CI = 0.22 to 0.76, Figure 2), 40 m sprint (r = −0.49, CI = −0.73 to −0.15, Figure 3), CMJ (r = 0.37, CI = 0.01 to 0.65), squat (r = −0.40, CI = −0.67 to −0.04), and bench press (r = −0.52, CI = −0.75 to −0.19) significantly correlated with Dist. Z46 in matches. The 12 min run did not show a significant correlation with Dist. Z46 in either BK or FW groups (Figure 4).

To further understand the relationship between physical characteristics (height, weight, %Fat, %Muscle, and BMI) and running performance in the match, partial correlation analysis was used with physical characteristics as an adjustment variable. As a result, RSA significantly correlated with Dist. Z46 in the match (r = 0.63, CI = 0.29 to 0.83) only in BKs. In FWs, there was a significant correlation between RSA and the 12 min run (r = 0.51, CI = 0.18 to 0.74) after the adjustment with physical characteristics.

## 4. Discussion

During an 80 min rugby match, players typically cover a distance of approximately 5 to 7 km [3,4,6]. Notably, high-intensity running, involving sprints at speeds equal to or exceeding 18.0 km/h, is predominantly observed during crucial moments of the game [5]. To delve into the relationship between physical characteristics, fitness factors, and high-intensity running, as represented by Dist. Z46, we performed separate studies focusing on backline (BK) and forward (FW) players. This division allowed us to consider their unique physical differences, ensuring a comprehensive understanding of the factors influencing high-intensity performance in rugby matches. By conducting these distinct investigations, we aimed to gain valuable insights that can be applied to tailor training programs and enhance the overall performance of both player groups on the rugby field.

### 4.1. For Backs

For BK players, we found a significant correlation between Dist. Z46 and %Fat and %Muscle. As previously reported [15], higher weight, fat mass, and %Fat correlated with lower endurance in rugby players. Additionally, lowering the %Fat enhances the endurance capacity of rugby players [16]. Considering the effect of endurance factors on the run distance covered in high-intensity running, our findings suggest that a lower %Fat and a higher percentage of body muscle would benefit Dist. Z46 during a match. To further understand the effects of physical characteristics (height and weight, %Fat, %Muscle, and BMI) on running performance in the match, partial correlation analysis was used with physical characteristics as an adjustment variable. As a result, RSA significantly correlated with Dist. Z46 in the match (r = 0.63) in BKs. This shows that the ability for Dist. Z46 during a match is better reflected by RSA.

Regarding each physical fitness test, Dist. Z46 was found to correlate highly with RSA. However, no correlations were found for the 12 min run or 40 m sprint. These results suggest that high Dist. Z46 revealed the excellent capacity to repeat sprints (i.e., RSA) in BKs. In addition, no correlation was found between Dist. Z46 and elevated blood lactate after the RSA. The average change in blood lactate after the RSA test was 16.1 ± 3.5 mmol/L. The RSA test involves repetitive sprint exercises that depend on glycolytic metabolic capacity. Therefore, the proportion of blood lactate after the RSA test was expected to increase in the subjects with better RSA. However, no correlations were found in the changes in blood lactate and with 40 m sprint, RSA, Dist. Z46, or Max. speed. It is speculated that the lactate increases during sprinting and decreases during active recovery [17]. Players with high Dist. Z46 have high sprint capacity in the match, thus lactate levels increase. On the other hand, these players are also presumed to have high aerobic capacities and can therefore remove lactate effectively [18,19]. The blood lactate level reflects the balance between the production and removal of lactate in muscle cells. In other words, it is conceivable that players with superior glycolytic and aerobic capacity also had superior Dist. Z46 during the match because of their superior RSA. The lack of correlation between lactate accumulation and RSA capacity suggests that other metabolic routes than lactate metabolism are key players in the RSA test.

In examining the relationship between Dist. Z46 and other running performance indexes during the match, we observed a significant correlation between Dist. Z46 and both Dist. and Dist. Z1. These correlations suggest that the distance covered in high-intensity running has an impact on the overall distance covered as well as on low-intensity running. Our findings lead us to speculate that players with greater high-speed running capacity also possess a solid foundation of endurance, as reflected in Dist. and Dist. Z1 during the match.

Regarding muscle strength and power, our study did not find a significant correlation with Dist. Z46 for BK players. This observation suggests that high-intensity running may not be heavily influenced by these specific attributes in the context of BK gameplay. However, it is essential to note that muscle strength and power still hold significant value in the sport, especially when it comes to executing strong tackling maneuvers.

To comprehensively gauge the relevance of muscle strength and power in BK players, it would be prudent to explore alternative ways of measuring field performance. Incorporating various performance metrics, such as agility, explosive movements, and tackling efficiency, could provide a more holistic understanding of how muscle strength and power impact BK players’ overall gameplay.

By integrating these additional performance indicators, coaches and sports scientists can gain deeper insights into the multi-faceted aspects of BK players’ physical capabilities. This enhanced knowledge can then inform tailored training programs that address the specific needs of each player, ultimately leading to improved on-field performance and strategic advantage during competitive matches.

### 4.2. For Forwards

In FW players, the Dist. Z46 correlated with %Muscle (r = 0.55) and had anti-correlations with the weight (r = −0.67), %Fat (r = −0.60), and BMI (r = −0.62). Oversize is a limiting factor for the high-intensity running ability during a match. However, it is particularly difficult for FWs to reconcile the opposing abilities of weight and high-intensity running, as a high weight is essential for scrum and tackling. Ideally, the weight must be high and body size must be large while maintaining a high-intensity running ability.

Moreover, our analysis revealed a significant correlation between Dist. Z46 and RSA (r = 0.54), indicating that players with greater Dist. Z46 scores tend to exhibit better repeated sprint ability. Additionally, we observed an intriguing anti-correlation between Dist. Z46 and the 40 m sprint time (r = −0.49). This suggests that FW players, similar to BK players, must possess not only high sprinting abilities but also substantial endurance capacity, which becomes crucial for executing repeated sprints and covering longer total running distances at high intensity during a match.

These findings underscore the multi-faceted nature of athletic performance in FW players and emphasize the importance of developing both sprinting prowess and endurance capabilities to excel in their sport. Understanding these interrelated factors can aid coaches and trainers in devising targeted training regimens to optimize the players’ overall performance on the field.

Dist. Z46 also had a significant correlation with CMJ (r = 0.37), squat (r = −0.40) and bench press (r = −0.52). The positive correlation with CMJ with Dist. Z46 was expected because CMJ reflects not only strength but also muscle contraction speed during muscle power production. In contrast, squats and bench presses reflect only muscle strength. Therefore, squat and bench press correlated negatively with Dist. Z46. These findings suggest that power is beneficial to sprinting but strength is not so important for Dist. Z46.

As squats and bench presses correlated negatively with Dist. Z46, high muscular strength in rugby players may be detrimental to high-intensity running ability in a game. However, high muscle strength is essential for a rugby player, and the interpretation of the relationship between muscle strength and running ability in a match needs further analysis.

The main limitations of this study are the small number of subjects and the possibility of in-game coincidences since the measurements were made during only two games. This makes it difficult to generalize the results of this study.

## 5. Conclusions

In conclusion, the results of this study showed that physical characteristics such as heavy weight and high %Fat have a negative correlation with running ability in both BKs and FWs in games. Moreover, we find a strong correlation of RSA with Dist. Z46 in BK and FW players, suggesting that RSA test performance is a relevant method to evaluate physical capacity out of the field.

These results underscore the importance of using the RSA test as a reliable and relevant method to assess players’ physical capacity beyond the confines of the field. By incorporating such assessments, coaches and trainers can gain valuable insights into the players’ overall fitness levels and tailor training programs accordingly, maximizing their potential on the field. Ultimately, our study emphasizes the need to consider not only technical skills but also physical attributes when striving for peak performance in sports.

## Figures and Tables

**Table 1:** Comparison of physical characteristics and/or physical fitness and running ability during the game between backs and forwards. BK: backs; FW: forwards; %Fat: percent body fat (%); %Muscle: percent body muscle (%); BMI: body mass index; RSA: repeated sprint ability (m); La: blood lactate (mmol/L); CMJ: countermovement jump (cm); Dist.: total distance run at all speeds (m); Dist. Z1: distance run at ≤5.4 km/h (m); Dist. Z23: distance run at 5.5–17.9 km/h (m); Dist. Z46: distance run at 18.0 km/h ≤(m); Max. speed (km/h): maximum run speed (km/h); Acc.: the number of accelerations 2.5 m/s^2^ ≤(times).

	BK			FW		
Height (cm)	172.7	±	4.5	175.8	±	5.0 *
Body mass (kg)	80.3	±	4.6	96.1	±	7.4 **
% Fat (%)	15.5	±	2.2	19.8	±	4.9 **
% Muscle (%)	80.2	±	2.1	75.4	±	3.1 **
BMI (kg/m^2^)	26.9	±	1.3	31.1	±	2.5 **
RSA (m)	640.4	±	12.6	607.4	±	23.2 **
La before RSA (mmol/L)	1.1	±	0.2	1.2	±	0.2
La after RSA (mmol/L)	17.2	±	3.6	15.0	±	3.4
Δ La	16.1	±	3.5	13.8	±	3.3
12 min. run (m)	2989.8	±	140.5	2750.3	±	171.3 **
40 m sprint (m)	5.41	±	0.13	5.71	±	0.3 **
CMJ (cm)	58.2	±	5.1	48.1	±	7.0 **
Squat (kg)	166.5	±	19.2	184.3	±	16.2 **
Bench press (kg)	110.9	±	13.4	119.5	±	10.7 *
Clean (kg)	96.1	±	12.1	102.5	±	12.0
Dist. (m)	7093.7	±	497.1	6332.7	±	438.4 **
Dist.Z1 (m)	1990.9	±	237.4	1734.8	±	256.1 **
Dist.Z23 (m)	4226.0	±	518.9	4108.1	±	530.5
Dist.Z46 (m)	876.9	±	236.6	489.9	±	246.3 **
Max. speed (m/s)	8.06	±	0.49	7.25	±	0.6 **
Acc. (t)	30.7	±	10.2	19.8	±	10.9 **

Significantly different between BK and FW * *p* < 0.05, ** *p* < 0.01.

**Table 2:** Correlation coefficients between physical characteristics and/or physical fitness and running ability during game in backs. %Fat: percent body fat; %Muscle: percent body muscle; BMI: body mass index; RSA: repeated sprint ability; La: blood lactate; CMJ: countermovement jump; Dist.: total distance run at all speeds; Dist. Z1: distance run at ≤5.4 km/h; Dist. Z2: distance run at 5.5–17.9 km/h; Dist. 46: distance run at ≤18.0 km/h; Max. speed: maximum run speed; Acc.: the number of accelerations ≤ 2.5 m/s^2^. Δmeans the amount of changes in blood lactate before and after the RSA test. * *p* < 0.05, ** *p* < 0.01.

																		Acc.
																	Max.	
																Z46		
															Z23			
														Z1	−0.61 ** −0.61 ** 0.55 **	0.68 **		
													Dist.		0.64 **	0.55 **		
												Clean						
											Bench press (kg)	0.64 **						
										Squat		0.54 *						
									CMJ			0.54 *						0.51 *
								Δ La										
							RSA									0.66 **		
						12min. min. run												
					40 m sprint												−0.48 *	
				BMI						0.56 **			−0.54 **		−0.47 *			
			Muscle	−0.72 **									0.63 **			0.46 *		
		Fat	−1.00 **	−0.72 ** 0.72 **									−0.63 **			−0.47 *		
	Mass mass	0.49 *	−0.48 *	0.52 *									−0.49 *		−0.58 *			
Height height	0.61 **																	
Body height (cm)	Body mass (kg)	% Fat (%)	% Muscle (%)	BMI (kg/m^2^)	40 m sprint (m)	12 min. run (m)	RSA (m)	Δ La mmol/L	CMJ (cm)	Squat (kg)	Bench press (kg)	Clean (kg)	Dist. (m)	Dist. Z1 (m)	Dist. Z23 (m)	Dist. Z46 (m)	Max. speed (m/s)	Acc. (t)

**Table 3:** Correlation coefficients between physical characteristics and/or physical fitness and running ability during game in forwards. %Fat: percent body fat; %Muscle: percent body muscle; BMI: body mass index; RSA: repeated sprint ability; La: blood lactate; CMJ: countermovement jump; Dist.: total distance run at all speeds; Dist. Z1: distance run at ≤5.4 km/h; Dist. Z2: distance run at 5.5–17.9 km/h; Dist. 46: distance run at ≤18.0 km/h; Max. speed: maximum run speed; Acc.: the number of accelerations ≤ 2.5 m/s^2^. * *p* < 0.05, ** *p* < 0.01.

																		Acc.
																	Max.	0.49 **
																Z46	0.57 **	0.72 **
															Z23			
														Z1	−0.79 **	0.49 **		
													Dist.		0.61 **	0.56 **		0.53 **
												Clean						
											Bench press (kg)		−0.40 *			−0.52 **	−0.49 *	
										Squat	0.54 **		−0.52 **			−0.40 *	−0.56 **	−0.45 *
									CMJ	−0.44 *							0.61 **	0.42 *
								Δ La										
							RSA		0.65 **	−0.62 **			0.49 **			0.54 **	0.56 **	0.49 **
						12min. min. run	0.78 **		0.49 **	−0.41 *			0.58 **					
					40 m sprint	−0.60 **	−0.80 **		−0.74 **	0.51 **	0.40 *	−0.44 *				−0.49 **	−0.70 **	−0.42 *
				BMI	0.80 **	−0.66 **	−0.86 **		−0.67 **	0.67 **	0.48 *		−0.51 **			−0.62 **	−0.72 **	−0.57 **
			Muscle	−0.85 ** −0.67 **	−0.67 **	0.54 **	0.54 **		0.62 **	−0.48 *				0.42 *		0.59 **	0.55 **	0.62 **
		Fat	−0.59 **	0.49 ** 0.49 ** 0.72 **	0.48 **	−0.40 *	−0.58 ** −0.58 **			−0.58 **						−0.42 *	−0.45 *	
	Mass mass		−0.66 **	0.74 **	0.52 **	−0.41 *	−0.57 **		−0.49 ** 0.60 **	0.60 **	0.39 * −0.50 **		−0.50 **			−0.67 **	−0.56 **	−0.75 **
Height height				−0.39 * −0.40 *	−0.40 *		0.43 *	0.49 *				0.52 **						
Body height (cm)	Body mass (kg)	% Fat (%)	% Muscle (%)	BMI (kg/m^2^)	40 m sprint (m)	40 m sprint (m)	RSA (m)	Δ La mmol/L	CMJ (cm)	Squat (kg)	Bench press (kg)	Clean (kg)	Dist. (m)	Dist. Z1 (m)	Dist. Z23 (m)	Dist. Z46 (m)	Max. speed (m/s)	Acc. (t)

## Data Availability

The data used to support the findings of this study are available from the corresponding author upon request.

## References

[1] Austin D., Gabbett T., Jenkins D. (2011). Repeated high-intensity exercise in professional rugby union. J. Sports Sci..

[2] Bailey S.J., Wilkerson D.P., DiMenna F.J., Jones A.M. (2009). Influence of repeated sprint training on pulmonary O_2_ uptake and muscle deoxygenation kinetics in humans. J. Appl. Physiol..

[3] Barr M.J., Sheppard J.M., Gabbett T.J., Newton R.U. (2014). Long-Term Training-Induced Changes in Sprinting Speed and Sprint Momentum in Elite Rugby Union Players. J. Strength Cond. Res..

[4] Brooks J.H.M., Kemp S.P.T. (2008). Recent Trends in Rugby Union Injuries. Clin. Sports Med..

[5] Cahill N., Lamb K., Worsfold P., Headey R., Murray S. (2013). The movement characteristics of English Premiership rugby union players. J. Sports Sci..

[6] Curran O.A., Carling C. (2018). Effect of the 2016 international rugby union trial law amendments on the physical running demands of rugby union games: A pilot dtudy. J. Aust. Strength Cond..

[7] Duthie G., Pyne D., Hooper S. (2003). Applied Physiology and Game Analysis of Rugby Union. Sports Med..

[8] Fitzsimons M., Dawson B., Ward D., Wilkinson A., Dawson-Hughes B., Ware D., Wilkinson A., Fitzsimons M. (1993). Cycling and running tests of repeated sprint ability. Aust. J. Sci. Med. Sport.

[9] Lacome M., Piscione J., Hager J.-P., Carling C. (2016). Analysis of Running and Technical Performance in Substitute Players in International Male Rugby Union Competition. Int. J. Sports Physiol. Perform..

[10] Leger L.A., Lambert J. (1982). A maximal multistage 20-m shuttle run test to predict VO_2_ max. Eur. J. Appl. Physiol. Occup. Physiol..

[11] Nalbandian H.M., Radak Z., Takeda M. (2017). Active Recovery between Interval Bouts Reduces Blood Lactate While Improving Subsequent Exercise Performance in Trained Men. Sports.

[12] Nicholas C.W. (1997). Anthropometric and physiological characteristics of rugby union football players. Sports Med..

[13] Posthumus L., Macgregor C., Winwood P., Darry K., Driller M., Gill N. (2020). Physical and Fitness Characteristics of Elite Professional Rugby Union Players. Sports.

[14] Quarrie K.L., Hopkins W.G., Anthony M.J., Gill N.D. (2013). Positional demands of international rugby union: Evaluation of player actions and movements. J. Sci. Med. Sport.

[15] Reardon C., Tobin D.P., Delahunt E. (2015). Application of Individualized Speed Thresholds to Interpret Position Specific Running Demands in Elite Professional Rugby Union: A GPS Study. PLoS ONE.

[16] Roberts S.P., Trewartha G., Higgitt R.J., El-Abd J., Stokes K.A. (2008). The physical demands of elite English rugby union. J. Sports Sci..

[17] Scott T.J., Delaney J.A., Duthie G.M., Sanctuary C.E., Ballard D.A., Hickmans J.A., Dascombe B.J. (2015). Reliability and Usefulness of the 30-15 Intermittent Fitness Test in Rugby League. J. Strength Cond. Res..

[18] Smart D., Hopkins W.G., Quarrie K.L., Gill N. (2014). The relationship between physical fitness and game behaviours in rugby union players. Eur. J. Sport Sci..

[19] Wadley G., Rossignol P.L. (1998). The relationship between repeated sprint ability and the aerobic and anaerobic energy systems. J. Sci. Med. Sport.

